# TP63 truncating mutation causes increased cell apoptosis and premature ovarian insufficiency by enhanced transcriptional activation of CLCA2

**DOI:** 10.1186/s13048-024-01396-2

**Published:** 2024-03-25

**Authors:** Yali Fan, Shuya Chen, Chunfang Chu, Xiaodan Yin, Jing Jin, Lingyan Zhang, Huihui Yan, Zheng Cao, Ruixia Liu, Mingwei Xin, Lin Li, Chenghong Yin

**Affiliations:** 1 Central Laboratory, Beijing Obstetrics and Gynecology Hospital, Capital Medical University, Beijing Maternal and Child Health Care Hospital, Beijing, 100006 China; 2 Department of Gynecology, Beijing Obstetrics and Gynecology Hospital, Capital Medical University, Beijing Maternal and Child Health Care Hospital, Beijing, 100026 China; 3 Department of Traditional Chinese Medicine, Beijing Obstetrics and Gynecology Hospital, Capital Medical University, Beijing Maternal and Child Health Care Hospital, Beijing, 100026 China; 4 Department of Gynecological Endocrinology, Beijing Obstetrics and Gynecology Hospital, Capital Medical University, Beijing Maternal and Child Health Care Hospital, Beijing, 100026 China; 5 Department of Gynaecology and Obstetrics, Beijing Friendship Hospital, Capital Medical University, Beijing, 100050 China; 6 Department of Obstetrics, Beijing Obstetrics and Gynecology Hospital, Capital Medical University, Beijing Maternal and Child Health Care Hospital, Beijing, 100026 China; 7 Department of Laboratory Medicine, Beijing Obstetrics and Gynecology Hospital, Capital Medical University, Beijing Maternal and Child Health Care Hospital, Beijing, 100026 China

**Keywords:** Premature ovarian insufficiency, TP63, CLCA2, Apoptosis, Whole-exome sequencing

## Abstract

**Background:**

Premature ovarian insufficiency (POI) is a severe disorder leading to female infertility. Genetic mutations are important factors causing POI. TP63-truncating mutation has been reported to cause POI by increasing germ cell apoptosis, however what factors mediate this apoptosis remains unclear.

**Methods:**

Ninety-three patients with POI were recruited from Beijing Obstetrics and Gynecology Hospital, Capital Medical University. Whole-exome sequencing (WES) was performed for each patient. Sanger sequencing was used to confirm potential causative genetic variants. A minigene assay was performed to determine splicing effects of *TP63* variants. A *TP63*-truncating plasmid was constructed. Real-time quantitative PCR, western blot analyses, dual luciferase reporter assays, immunofluorescence staining, and cell apoptosis assays were used to study the underlying mechanism of a TP63-truncating mutation causing POI.

**Results:**

By WES of 93 sporadic patients with POI, we found a 14-bp deletion covering the splice site in the *TP63* gene. A minigene assay demonstrated that the 14-bp deletion variant led to exon 13 skipping during TP63 mRNA splicing, resulting in the generation of a truncated TP63 protein (TP63-mut). Overexpression of TP63-mut accelerated cell apoptosis. Mechanistically, the TP63-mut protein could bind to the promoter region of *CLCA2* and activate the transcription of *CLCA2* several times compared to that of the TP63 wild-type protein. Silencing CLCA2 using a specific small interfering RNA (siRNA) or inhibiting the Ataxia Telangiectasia Mutated (ATM) pathway using the KU55933 inhibitor attenuated cell apoptosis caused by TP63-mut protein expression.

**Conclusion:**

Our findings revealed a crucial role for CLCA2 in mediating apoptosis in POI pathogenesis, and suggested that CLCA2 is a potential therapeutic target for POI.

**Supplementary Information:**

The online version contains supplementary material available at 10.1186/s13048-024-01396-2.

## Background

Premature ovarian insufficiency (POI) is a prevalent infertility disease affecting females, with an estimated prevalence of 1–5% [1, 2]. It is characterized by the loss of normal ovarian function in women under 40 years of age, resulting in infertility and a range of menopausal symptoms, such as amenorrhea, lasting more than 4–6 months. In patients with POI, the blood follicle-stimulating hormone (FSH) level increases to > 25 IU/L [3]. The pathogenesis of POI is not well known, and the mechanisms underlying POI have been proposed to include chromosomal or genetic alterations, infections, metabolic disorders, autoimmune diseases, and iatrogenic factors [4–6], with genetic factors accounting for about 20–25% of the total cases [2]. In recent years, high-throughput sequencing technology has enabled the analysis of genetic information in patients with POI, leading to the identification of pathogenic genes closely related to POI. Several genes have been associated with POI, such as *BRCA2* [7], *C14orf39* [8], *ZSWIM7* [9], *PSMC3IP* [10], *NHEJ1* [11], *HSF2BP* [12], *MSH4* [13], *MSH5* [14], *FIGLA* [15], *NOBOX* [16], *STAG3* [17], *MCM8/9* [18], *HFM1* [19], *EIF4ENIF1* [20], *KHDRBS1* [21], *NOTCH2* [22], *BNC1* [23]. These genes participate in various activities such as folliculogenesis, gonadogenesis, oocyte maturation, DNA damage, meiosis, cell apoptosis, and various other functions [24].

Recently, there has been renewed interest in *TP63* gene, which is related to human infertility [25]. An increasing number of reports have confirmed the role of TP63 in mouse and human reproduction. TP63 is indispensable for embryonic craniofacial, skin, and limb development [26]. Some human diseases, such as limb malformations, ectodermal dysplasia, craniofacial anomalies, and isolated POI, have been reported to be related to *TP63* [27]. Different studies have found that *TP63* missense or truncation mutations cause POI [28, 29]. The main function of *TP63* is to regulate the biological function of epithelial cells and oocytes [30, 31], which might be related to the expression level of TP63. As an important component of the p53 family of transcription factors, *TP63* is specifically expressed in female germ cells and is specific to oocytes [30, 32]. *TP63* is expressed either in cooperation or independent of p53, and protects genomic stability and maintains female reproductive function [33]. Studies have also reported the expression of *TAp63α* in male germ cells [34, 35].

The C-terminus of TP63 plays a major role in its function, and based on different C-terminal variants, *TP63* can be divided into five different subtypes. TAp63α contains the longest structure and most complete function [36]; the structure includes a basic N-terminal transcription activation domain (TAD), a unique sterile alpha motif domain (SAM), and a transcription inhibition domain (TID). The SAM domain is mainly involved in protein-protein interactions, but its specific function remains unknown [37, 38]. The TID domain is critical for the stable formation of TP63 dimeric complexes [39–41]. The SAM domain specifically regulates the transcription of exon 13. Studies found that exon 13-truncated POI mice exhibited more expression of TAp63β tetramers because TAp63β lacks the SAM structure domain responsible for encoding exon 13. Therefore, the SAM domain is important for *TP63* function, and the stable dimeric conformation of TP63 is an important factor for female reproductive health.

The role of TP63 in reproductive function is mainly reflected in the first meiotic division of primary oocytes. During homologous chromosome pairing, TP63 expression is relatively reduced under physiological conditions to maintain normal numbers and activity of dividing oocytes, and to resist oocyte death caused by DNA damage. TP63 is strictly regulated during this process. Under physiological conditions, TP63 maintains a non-active and closed dimeric conformation. However, in the case of pathological DNA damage, the ATM-CHK1/CHK2-CK cascade is activated; the TP63 structure changes to the opening state and is phosphorylated to an active tetrameric structure. This, in turn, induces oocyte apoptosis and leads to an excessive depletion of follicle numbers, resulting in the loss of normal reproductive function of the ovary [41–44]. In a mouse model, *TP63*-deficient oocytes were resistant to radiation or chemotherapy-induced DNA damage, and the cells were unable to recover normal function [30, 32]. Knocking down the *TP63* gene led to a severe reduction in the number and activity of primordial follicles, and induced a state of cellular senescence [45, 46]. These findings confirmed the critical role of *TP63* in follicular development.

However, the mechanisms by which *TP63* gene mutations lead to POI remain unclear. Based on previous reports, we conducted experiments and found that the *TP63* exon 13 truncation mutation in patients with POI did not exert its effects through the BAX pathway in the exon 13-deleted mouse model [41, 47]. Therefore, the mechanism by which *TP63* leads to cell apoptosis and subsequent induction of POI was the focus of the present study. Through RNA sequencing of cells transfected with a high expression of the *TP63*-truncating mutation, we found that *TP63-*truncating mutations abnormally activated the *CLCA2* promoter, elevating the expression of CLCA2 protein, and accelerating the process of cell apoptosis. When *CLCA2* was knocked down, the phenomenon of cell apoptosis was alleviated. Therefore, we hypothesize that TP63 regulation of *CLCA2* plays an important role in the progression of POI.

Here, through whole-exome sequencing (WES) of 93 patients with sporadic POI, we identified a 14-bp deletion mutation in the *TP63* gene in one patient with POI, suggesting that the 14-bp deletion may lead to the skipping of exon 13 during mRNA splicing, resulting in the generation of a truncated TP63 protein. Overexpression of the truncated TP63 protein caused a significant increase in apoptosis. RNA-sequencing (RNA-seq) analysis revealed that cells with truncated TP63 expression had significantly higher expression of the *CLCA2* gene compared to that of cells expressing wild-type TP63. Silencing *CLCA2* attenuated apoptosis. Therefore, CLCA2 is an important factor that mediates apoptosis caused by *TP63* mutations and may be a potential therapeutic target for the treatment of POI.

## Methods

### Patients

Ninety-three patients with POI were recruited from the Beijing Obstetrics and Gynecology Hospital, Capital Medical University from Mar 2021 to Apr 2023. POI was diagnosed if the patient had oligo/amenorrhea for at least 4 months, if the patients were below 40 years of age, and if two consecutive FSH measurements were > 25 IU/L, performed > 4 weeks apart. Patients with POI were excluded from the study if they showed any of the following: karyotypic abnormalities (X chromosome abnormalities), autoimmune disorders, history of radiotherapy and chemotherapy, or pelvic surgery. The information for this *TP63* mutation patient was showed at the Supplemental Table 1.

### Wes

WES was performed as described previously [48]. The criteria used for screening were missense, nonsense, frameshift, or splice site variations and variations with minor allele frequencies of < 1%. Second, allele frequency data were obtained by referring to the following databases: the Genome Aggregation Database (gnomAD, http://gnomad.broadinstitute.org/), the NHLBI Exome Sequencing Project (ESP6500), and the 1000 Genomes Project (1000G, http://browser.1000genomes.org/index.html). Sanger sequencing was used to validate the *TP63* mutations in patients with POI.

### Plasmid construction

#### Construction of plasmids for the minigene assay

We hypothesized that the c.1742_1749 + 9del variant might affect the splicing of exon 13 in the *TP63* gene, so a sequence containing exon 12, intron 12, exon13, intron 13, and exon 14 was constructed. A vector containing the wild-type genomic sequence was constructed as follows: PCR was used to amplify a 5508 base pair sequence containing exon 12, intron 12, exon 13, intron 13, and exon 14 with an ATG (start codon) and TGA (stop codon) sequence; additionally, a restriction enzyme sequence flanking the whole sequence was constructed into the pcDNA3.1 vector using the XhoI (5′) and BamHI (3′) restriction enzyme digestion sites. To construct the mutant plasmid containing the c.1742_1749 + 9del variant, overlapping PCR was used to introduce the c.1742_1749 + 9del mutational site into the WT sequence to obtain the mutant plasmid.

#### Construction of overexpression plasmids for TP63 wild-type and truncated proteins

The full-length human *TP63* coding sequence (NM_003722) was cloned into the pcDNA3.1–3× FLAG vector. A mutant sequence encoding a p.S551* TP63 truncated protein was also constructed in the pcDNA3.1–3 × Flag vector.

#### Construction of pGL3-*CLCA2* promotor plasmid

Through bioinformatics analysis and information from the websites https://www.genecards.org/ and https://genome.ucsc.edu/, a 500 bp sequence of the *CLCA2* promoter was determined as the region regulated by the *TP63* gene, and primers were designed. PCR was used to amplify the sequence containing the *CLCA2* promoter region, and a restriction enzyme sequence flanking the whole sequence was constructed into the reporter gene vector (pGL3-Basic plasmid) using the NheI (5′) and Xho1 (3′) restriction enzyme digestion sites. As a result, the pGL3-*CLCA2* promotor (pGL3-*CLCA2* prom.) plasmid was successfully constructed.

All The primers for the plasmid construction were showed at the Supplemental Table 2–3.

#### Cell culture and reagents

293FT cells were grown in Dulbecco’s Modified Eagle Medium (DMEM)/basic (1×) (C11995500BT, Gibco, USA) supplemented with 10% fetal bovine serum, GlutaMAX™-I (100×) (35050–061; Gibco, USA), MEM NEAA (100×) (11140–050; Gibco, USA), and Strep (15140–122; Gibco, USA) under 5% CO_2_ conditions. The ATM inhibitor (KU55933) was obtained from MCE (HY-12016; MCE, Monmouth Junction, NJ, USA).

#### Plasmid transfection

293FT cells were seeded in a six-well plate with 2.5 × 10^5^ cells per well. Cells were 20–40% confluent at the time of transfection. Transfection with jetPRIME (101,000,046; Polyplus, Illkirch, France) was perfomed according to the manufacturer’s instructions. Two micrograms of DNA were diluted into 200 μl jetPrime buffer and mixed by vortexing. Next, 4 μl jetPrime was added, vortexed for 10 s, spun down briefly, and the mixture was incubated for 10 min at 22 °C. Then, 200 μl of transfection mix was added to each well and distributed evenly. The plates were gently agitated, and if needed, the transfection medium was replaced after 4 h with cell growth medium before being returned to the incubator. The cells were collected for reverse transcription-quantitative polymerase chain reaction (RT-qPCR) and western immunoblotting after 48 h.

#### RT–qPCR

Total RNA was extracted using the HiPure Total RNA Mini Kit (R4111–03; Magen, China), and cDNA was synthesized using TransScript One-Step gDNA Removal and cDNA Synthesis SuperMix (AT311–03; TransGen Biotech, Beijing, China) according to the manufacturer’s protocol. RT-qPCR was performed using the PerfectStart Green qPCR Super Mix (AQ601–04; TransGen Biotech, Beijing, China) on a fluorescent RT-qPCR instrument (LightCycler 480 II; Roche, Basel, Switzerland). Relative gene expression levels were normalized to the critical threshold value of the housekeeping gene *ACTB*. The primers used for all sequences are listed in Table S2. RT-qPCR was performed in triplicates.

#### Western immunoblotting

293FT cells were transfected with plasmids for 48 h. Total cell lysates were prepared in RIPA buffer (R0020; Beijing solarbio science & technology co.,ltd. Beijing, China) containing cOmplete Protease Inhibitor Cocktail Tablets (04693124001; Roche, Germany). The protein concentration was quantified using a BCA assay (P0009; Beyotime Biotechnology, Shanghai, China). Equal amounts of lysates were electrophoresed on 10–12% SDS-PAGE and transferred onto a 0.2 μm Polyvinylidene Fluoride (PVDF) membrane (ISEQ00010; Merck Millipore Ltd., Tullagreen, Cork Ireland). The membranes were probed at 4 °C overnight with appropriate primary antibodies with 1:1000 dilutions. Antibodies against DYKDDDDK Tag (9A3) Mouse mAb (#8146; Cell Signaling Technology, MA, USA), and the CLCA2 polyclonal antibody (19273–1-AP; ProteinTech, Wuhan, China) were used. The membrane was then washed with 1 × TBST, followed by the addition of the secondary antibody for 1 h with anti-mouse IgG (H + L) biotinylated antibody (ZB-2305; Zhong Shan-Golden Bridge Biological Technology Co., LTD, Beijing, China) or anti-rabbit IgG (H + L) biotinylated antibody (ZB-2301; Zhong Shan-Golden Bridge Biological Technology Co., LTD, Beijing, China). Proteins were visualized using Immobilon Western HRP Substrate Luminol Reagent (WBKLS0500; Millipore, Billerica, MA, USA) and a ChemiDoc imaging system (Bio-Rad, Hercules, CA, USA).

#### Cell apoptosis assay

Apoptosis was determined using an Annexin V-EGFP Apoptosis Detection Kit (C1067M; Beyotime Biotechnology, Shanghai, China) following the manufacturer’s protocol. The cells were seeded in six-well plates at a density of 2.5 × 10^5^ cells/well and then treated with vehicle or plasmids for 48 h. The cells were harvested and stained with 100 μl of Annexin-V for 15 min in dark. Annexin-V expression was determined using FlowJo_v10.8.1 software (BD, Ashland, OR, USA).

#### RNA sequencing and data analysis

RNA-seq was performed using Berry Genomics (Beijing, China). Total RNA was extracted as described above, and RNA quality was analyzed using a Agilent 2100 Bioanalyzer (Agilent Technologies, Palo Alto, CA, USA). The cDNA libraries were constructed using the Illumina NovaSeq 6000 sequencing platform (San Diego, USA). Samples from three biological replicates were analyzed. Eukaryotic mRNA enrichment was performed using magnetic beads with Oligo (dT); mRNA was broken into short segments, the first strand of cDNA was synthesized using the segmented mRNA as a template, and then the second cDNA strand was synthesized by adding buffer solution, dNTPs, and enzymes. The obtained double-stranded cDNA was purified, poly-A was added, the fragment was selected, and the cDNA libraries were enriched. A Qubit 3.0 fluorimeter was used for preliminary quantification, and qPCR was used for accurate quantification. These reads were filtered to obtain clean high-quality reads for gene expression and structural analysis. Genes that showed a > 2-fold difference (FC > 2) and *p* < 0.05 were selected for further analysis. Gene Ontology (GO) analysis was performed using the gene annotation and analysis resource Metascape (http://metascape.org/gp/ index.html).

#### Small interfering RNA (siRNA) transfection

*CLCA2* knockdown was performed by transfecting specific siRNA (Supplementary Table 3) in 293FT cells (2.5 × 10^5^ cells/well) for 48 h. Cells were transfected with siRNA using jetPrime (101,000,046; Polyplus, Illkirch, France) according to the manufacturer’s protocol. The non-specific siRNA vector was used as a scramble, and the cells were cultured for 48 h for the later assays.

#### Luciferase assay

A dual-luciferase reporter assay kit was obtained from Vazyme Biotech Co., Ltd. (DL101–01; Nanjing, China). 293FT cells were seeded into 24-well plates, and when the cells were 20–40% confluent, the plasmid was transfected into the cells for 48 h. Next, the cell culture medium was discarded, cells were washed twice with PBS, and 1 × cell lysis buffer was added for 5 min at 22 °C. Cells were then centrifuged at 12,000×g for 2 min at 22 °C, and the supernatant was collected for subsequent detection. Twenty microliters of cell lysate supernatant were added to the enzyme in a standard 96-well plate, followed by the addition of 100 μl of luciferase substrate into each well. This solution was quickly mixed, and the firefly luciferase gene activity was immediately detected using GloMax Discover System (Promega, Madison, WI, USA). Next, 100 μl of Renilla subtract working solution (freshly prepared) was added to the reaction solution, quickly mixed, and then Renilla luciferase activity was immediately detected using GloMax Discover System (Promega, Madison, WI, USA).

#### Statistics and reproducibility

Statistical analyses were performed using GraphPad Prism (version 8). One-way analysis ANOVA was used to compare the two sets of data with an assumed normal distribution. And the Bonferroni Multiple Comparison Test fort the post hoc test to correct the number of comparisons. The data was presented by the mean ± SD from at least three independent experiments. In statistics, the * denotes *p* < 0.05, ** and *** mean *p* < 0.01 and *p* < 0.005, respectively, all of which were considered to be statistically significant. All representative experiments were independently repeated at least three times.

## Results

### Identification of *TP63* variant and the splicing effect of the 14-bp deletion

**Fig. 1:**
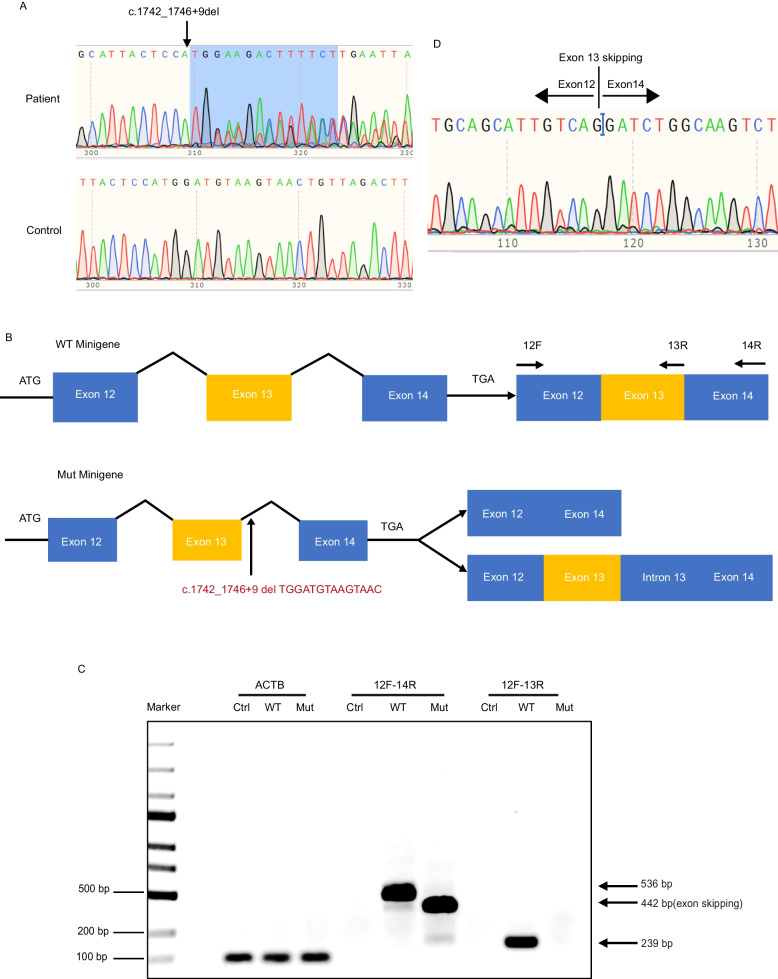
*TP63* variant form and the splicing effect of the 14-bp deletion (**A**) Sanger sequencing validated the *TP63* variant in the patient with POI. The *red arrow* indicates the variant c.1742_1746 + 9del. (**B**) The variant influenced the mRNA splicing of the exon 13 and intron 13, so the WT minigene plasmid containing exon 12, intron 12, exon 13, intron 13, and exon 14 of the *TP63* gene was constructed, and then the c.1742_1749 + 9del 14-bp deletion variant was introduced into the WT plasmid to obtain the mutational plasmid (Mut minigene). (**C**) The pcDNA3.1 empty vector (NC), WT, and Mut minigene plasmids were transfected into 293FT cells, and then the RNA was collected for the RT-PCR. The forward primer in exon 12 (12F), the reverse primer in exon 14 (14R), the forward primer in exon 12 (12F), and the reverse primer in exon 13 (13R) were used. The RT-PCR results indicated that 442 -bp bands were compatible with exon 13 skipping under Mut minigene conditions, while in the WT minigene, 536-bp bands corresponding to the correctly spliced product obtained using 12F and 14R primer pairs. For the 12F and 13R primer pairs, a 239-bp band product was obtained, corresponding to the correctly spliced product under WT minigene condition. There was no band compatible with exon 13 skipping under Mut minigene conditions. We conclude that the RT-PCR obtained represented the Mut minigene performed as the exon-12–14 transcript. (**D**) The cells transfected with Mut minigene plasmids were tested by Sanger sequencing, and the results showed the 14-bp deletion variant induced exon 13 skipping

93 patients diagnosed with POI were recruited for this study. WES was performed on each patient with POI. A 14-bp heterozygous deletion variant, c. 1742_1746 + 9del, in the *TP63* gene was found in one patient with POI. The hormone levels of this patient are listed in Supplemental Table 1. Sanger sequencing was performed to validate the variant (Fig. 1A). This variant covers the boundary region of exons and introns, which may affect mRNA splicing. We used a minigene assay to determine the splicing effect of the 14-bp deletion. A WT minigene plasmid containing exon 12, intron 12, exon 13, intron 13, and exon 14 of the *TP63* gene was constructed (Fig. 1B). Additionally, the 14-bp deletion variant c.1742_1749 + 9del was introduced into the WT plasmid to obtain a mutant plasmid (Mut minigene).

Subsequently, the pcDNA3.1 empty vector, WT, and Mut minigene plasmids were transfected into 293FT cells. WT minigene plasmid-expressing cells can splice introns and form a normal exon 12–13-14 transcript. However, mut-minigene plasmid-expressing cells predominantly spliced the entire sequence of intron-12-exon-13-intron-14, resulting in an exon-12–14 transcript (Fig. 1C). The skipped exon 13 in the Mut minigene plasmid-expressing cells was further validated by Sanger sequencing (Fig. 1D). Therefore, the minigene assay demonstrated that the 14-bp deletion variant led to exon 13 skipping, which was predicted to generate a TP63-truncated protein p.S551Rfs*6.

### The TP63-truncated protein promoted cell apoptosis

**Fig. 2:**
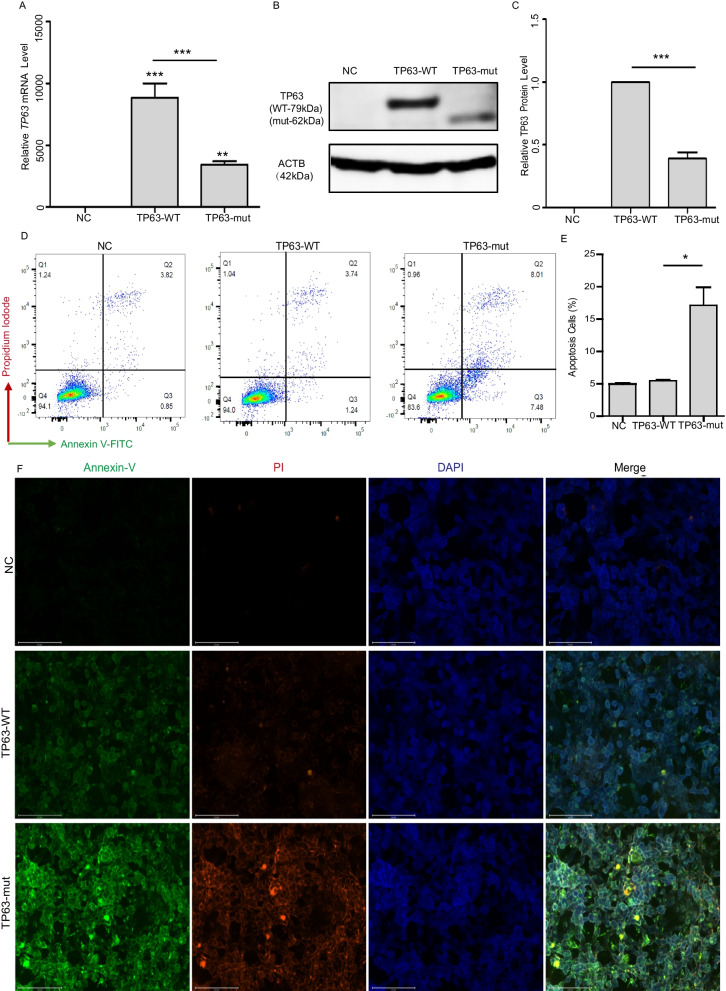
TP63-truncated protein accelerated the cell apoptosis (**A**) *TP63* exon 13 skipping resulted in the generation of a TP63-truncated protein p.S551Rfs*6. The plasmid expressing a p.S551* A TP63-truncated protein was constructed (TP63-mut). The empty vector (NC), TP63-WT, and TP63-mut were transfected into 293FT cells. The RT-qPCR results showed that the relative *TP63* mRNA expression in TP63-mut was lower than that of the TP63-WT, and the ***p < 0.001. (**B-C**) The western immunoblotting assay showed that the expression of TP63 in TP63-mut was less than that of the TP63-WT. (**D-E**). The TP63-truncated protein accelerated apoptosis; Annexin-V was analyzed using Flowjo_v10.8.1 and GraphPad Prism. (**F**) The morphology of cells stained with Annexin-V, PI and DAPI was observed under a fluorescence microscope with a scale bar of 125 μm. The TP63-truncated protein accelerated apoptosis.The TP63-mut enhanced cell apoptosis

To study the role of the TP63-truncated protein, a plasmid expressing the p.S551* TP63-truncated protein (TP63-mut) was constructed. The same amount of TP63 wild-type (TP63-WT), TP63-mut, or empty vector (NC) was transfected into 293FT cells, and we found that TP63-mut was expressed at lower levels than TP63-WT at both the mRNA and protein levels (Fig. 2A, B and C). We further elucidated the functional impact of the TP63-truncated protein. Considering that TP63 is reported to be associated with cell apoptosis, an Annexin V assay was carried out. We found that 15.49% of TP63-mut expressing cells were apoptotic; however, only 4.98% of TP63-WT expressing cells were apoptotic (Fig. 2D and E), suggesting that the TP63-mut protein increased cell apoptosis. Immunostaining of Annexin V and PI in TP63-mut and TP63-WT expressing cells also demonstrated that the TP63-mut protein enhanced cell apoptosis (Fig. 2F).

### RNA-seq analysis revealed *CLCA2* to be a downstream target gene of the TP63-Mut protein

**Fig. 3:**
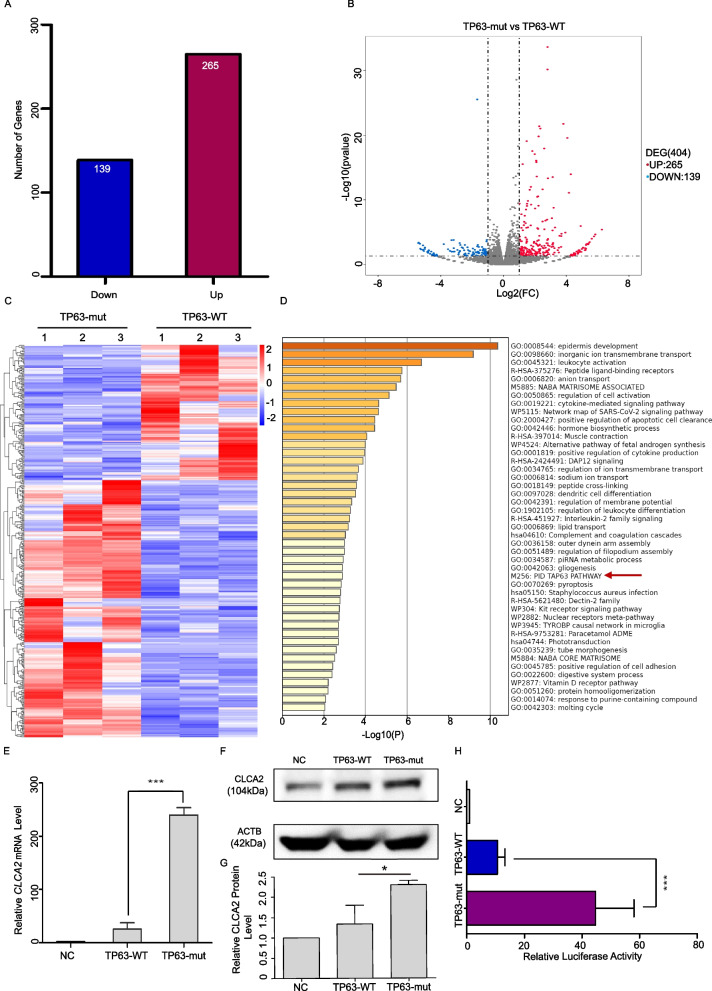
The TP63-truncated protein activated additional expression of *CLCA2*, demonstrating that *CLCA2* was the downstream target gene (**A–C**) The results of RNA-seq showed that there were 404 genes differentially expressed between TP63-WT and TP63-Mut, of which 265 genes were increased and 139 genes were decreased ((*p* < 0.05) and (|log2FC| > 1)). (**D**) GO analyses of TP63-WT and TP63-Mut genes with increased expression (Top: *p* < 0.05). (**E–G**) The mRNA level of *CLCA2* (***p < 0.001) was higher in TP63-Mut than that in TP63-WT, and the western immunoblotting results showed greater expression of CLCA2 protein in TP63-Mut compared to that of TP63-WT. (**H**) The results of the luciferase assay showed that TP63-mut regulated the increased expression of the *CLCA2* promoter (*p* < 0.001), indicating that *CLCA2* was a downstream target gene of the TP63-truncated protein

To further elucidate the molecular mechanism by which TP63-mut induces apoptosis, RNA-seq analysis was performed. A total of 404 genes were differentially expressed between TP63-mut and TP63-WT expressing cells, of which 265 genes and 139 genes were expressed at higher or lower expression levels in TP63-mut expressing cells, respectively (Fig. 3A–C). Gene Ontology (GO) analysis of the 265 highly expressed genes suggested that the TP63 pathway was significantly enriched (Fig. 3D), indicating that the TP63-mut protein had higher gene transcriptional activity than the TP63-WT protein. Among the 265 highly expressed gene in TP63-mut expressing cells, *CLCA2* was the gene with the highest differential expression level, which was also validated by qPCR analysis (Fig. 3E). Western blotting confirmed that CLCA2 protein expression was higher in TP63-mut expressing cells (Fig. 3F and G). To further elucidate whether the TP63-mut protein could directly transactivate *CLCA2* gene expression, a luciferase assay was performed, and a direct and higher luciferase activity was found in TP63-mut expressing cells compared with that of TP63-WT expressing cells (Fig. 3H). Therefore, *CLCA2* is a downstream target of the TP63-mut protein.

### Knockdown of *CLCA2* decreased the cell apoptosis induced by the TP63-Mut protein

**Fig. 4:**
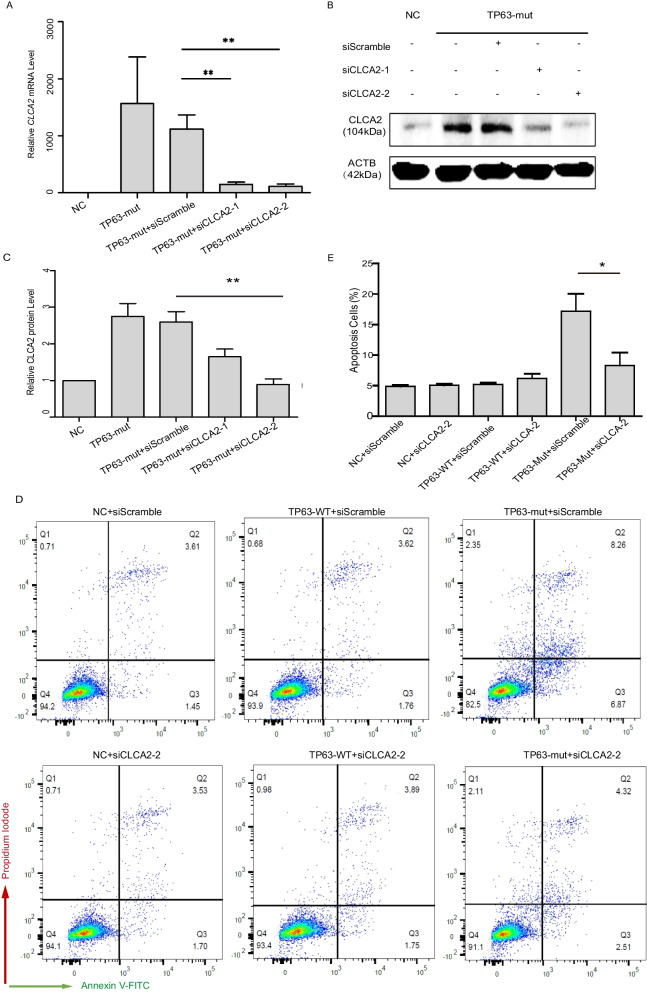
siCLCA2 decreased the cell apoptosis induced by the TP63-mut (**A**) The RT-qPCR data showed a significant decrease in CLCA2 expression upon treatment with siCLCA2–1 and siCLCA2–2 (*p* < 0.01). (**B-C**) The western immunoblotting assay revealed a significant decrease in CLCA2 expression following treatment with siCLCA2–2. (**D-E**) The Annexin-V assay showed that siCLCA2 inhibited cell apoptosis induced by TP63-mut

Next, to determine whether CLCA2 was involved in TP63-mut protein-induced cell apoptosis, specific siRNAs were used to knockdown *CLCA2* expression (Supplemental Table 2). qPCR showed that both siCLCA2–1 and siCLCA2–2 can decrease about 80% CLCA2 expression compared to the siScramble group in the context of TP63-mut overexpression (Fig. 4A). Western blot analysis showed that both siCLCA2–1 and siCLCA2–2 efficiently decreased CLCA2 protein expression (Fig. 4B and C). Surprisingly, siCLCA2 significantly decreased TP63-mut protein expression-induced cell apoptosis (Fig. 4D,E and Supplemental Fig. 1), suggesting that CLCA2 may be a TP63-mut downstream target gene that mediates cell apoptosis induced by TP63-mut protein expression.

### An ATM inhibitor can decrease *CLCA2* expression and inhibit cell apoptosis induced by TP63-Mut protein expression

**Fig. 5:**
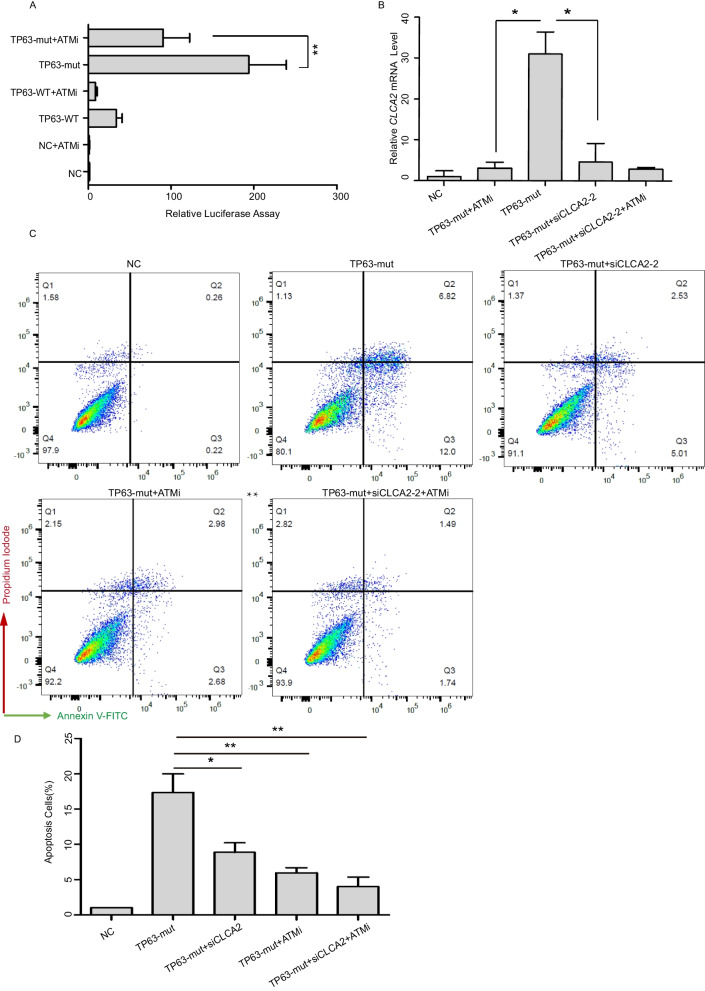
The ATM inhibitor decreased the expression of *CLCA2* and inhibited cell apoptosis induced by TP63-mut protein expression (**A**)293FT cells were transfected with empty vector (NC), TP63-WT, TP63-mut, pGL3-Basic-CLCA2 prom, and pRL-TK plasmids, and treated with the ATM inhibitor (ATMi). Luciferase assay results demonstrated that the expression of the CLCA2 promoter was significantly reduced in TP63-mut + ATMi cells compared to that of TP63-mut cells, indicating that the ATM inhibitor reduced CLCA2 expression to regulate the effect of TP63-mut protein. (**B**) 293FT cells were transfected with empty vector (NC), TP63-WT, TP63-mut, pGL3-Basic-CLCA2 prom, and pRL-TK plasmids, and treated with ATMi and siCLCA2. The RT-qPCR data further confirmed that ATMi inhibited CLCA2 expression, with a more pronounced effect observed in cells treated with both ATMi and siCLCA2. (**C-D**) The combination of ATMi and siCLCA2 inhibited the pro-apoptotic effect of the TP63-truncating mutation. Annexin-V was analyzed using Flowjo_v10.8.1 and GraphPad Prism

**Fig. 6:**
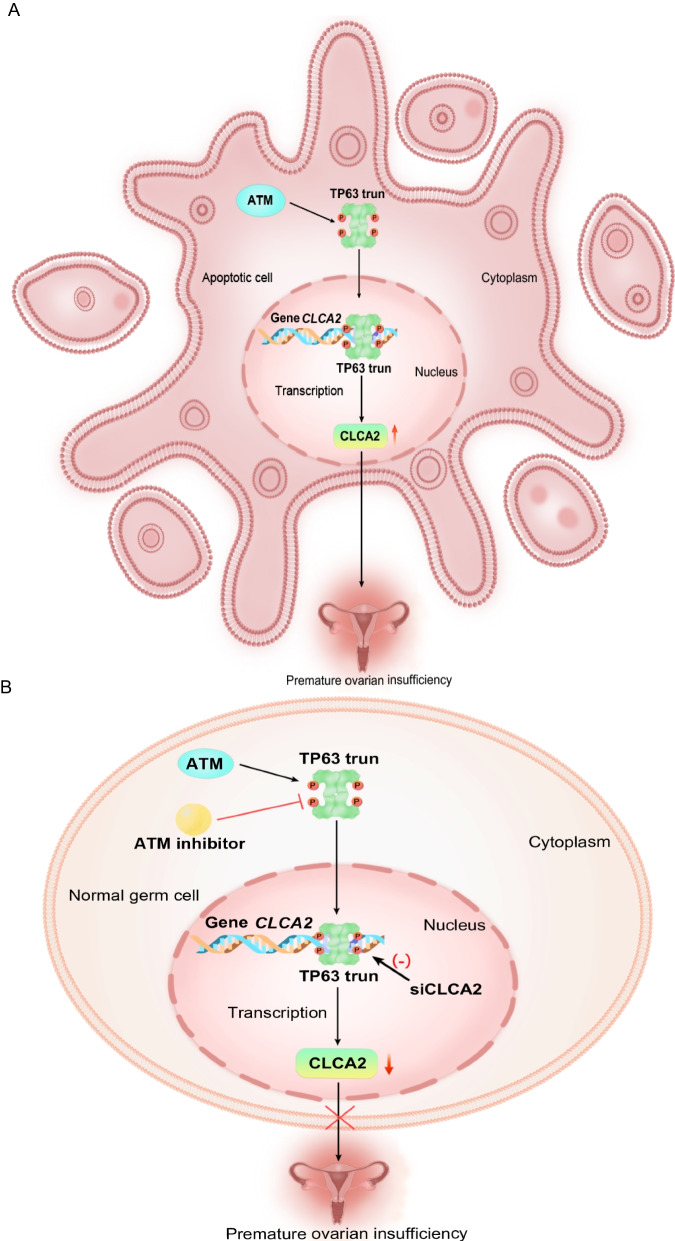
The mechanism of the *TP63-*truncating mutation leading to POI. (**A**) The ATM-TP63 pathway regulates TP63 activity in healthy ovarian cells. However, when the TP63-truncating variant (TP63 trun) was generated, it activated the downstream target gene *CLCA2*, thereby stimulating its transcription and expression. This, in turn, induced more cell apoptosis, ultimately leading to POI. (**B**) The silencing of *CLCA2* or the use of ATMi significantly inhibited the apoptotic effect of the TP63-truncated protein and decreased cell apoptosis

Recently, the ATM-TP63 pathway was shown to play a key role in the elimination of mouse oocytes induced by double-stranded DNA breaks (DSBs) [44]. It was reported that the ATM can phosphorylate TP63, and the use of an ATM inhibitor (ATMi) inhibited the tetramerization and activation of TP63 in a dose-dependent manner [42]. Therefore, we wanted to determine whether ATMi decreased apoptosis induced by the TP63-mut protein. Adding ATMi to the cell culture media significantly decreased *CLCA2* expression in the context of overexpressed TP63-mut protein (Fig. 5B). A luciferase assay also demonstrated that ATMi attenuated TP63-mut protein binding to the promotor of *CLCA2* (Fig. 5A). Annexin V assay and immunofluorescent staining of Annexin V suggested that ATMi treatment significantly decreased cell apoptosis induced by overexpression of the TP63-mut protein (Fig. 5C-D and Supplemental Fig. 2). Therefore, a potential model can be proposed: a TP63-truncated protein can induce cell apoptosis mediated by the transactivation of *CLCA2* (Fig. 6A), and silencing *CLCA2* using siCLCA2 or ATMi treatment can significantly decrease *CLCA2* expression and inhibit cell apoptosis induced by TP63-mut protein expression (Fig. 6B), indicating a potential treatment for patients with POI who harbor a TP63-truncating mutation.

## Discussion

In the present study, a novel truncating mutation of *TP63* was identified. Functional experiments demonstrated that the TP63-mut protein increased cell apoptosis via transcriptional activation of *CLCA2*. Specific knockdown of CLCA2 using siRNA or treatment with ATMi alleviated the pro-apoptotic effects of TP63-mut protein.

The *TP63* gene is widely expressed in female reproductive organs, with the highest expression in the vagina and ovaries [49, 50]. It plays a crucial role in balancing the functions of female reproductive cells at different stages of development, stabilizing normal meiotic division of oocytes, and ensuring ovarian health. Luan et al. observed the process of meiotic division in primitive reproductive cells of female mice and found that the expression level of TP63 decreased from embryonic day E13.5 to E16.5 during meiotic division. From E17.5 to postnatal day 5 (PD5), TP63 was highly expressed in primitive, primary, and early secondary follicles. This suggests that TP63 can maintain the orderly progress of the first meiotic division by regulating its own expression, which helps complete the second meiotic division and fertilization [25, 51]. It has also been discovered that the *TP63* C-terminus is important for the development and terminal differentiation of germ cells [25, 49], and that changes in the *TP63* C-terminus can affect the genomic integrity of female reproductive cells, ultimately affecting female fertility. However, the specific mechanisms through which TP63 regulates development remain unknown. Nevertheless, some evidence suggests that *TP63* forms oligomers through its C-terminal domain and negatively transactivates downstream target genes, thereby participating in the developmental process of reproductive cells [52, 53]. The TP63 C-terminus regulates the transcription of exons 12, 13, and 14 [54], which encode the TP63 protein that maintains its basic function in an inactive dimeric structure. However, under pathological conditions, it is phosphorylated into an active tetrameric structure, which exerts its pathological effects [55]. Studies have found that under radiation exposure, mouse ovaries exhibit a higher intensity of active tetrameric TP63 protein signal, whereas the concentration of TP63 in non-irradiated ovaries is significantly higher than that in irradiated ovaries. Experimental evidence has shown that during the homologous chromosome recombination period of meiotic division, if the intensity of DNA DSBs exceeds the normal range by 4–10 times, TP63 transforms into an active tetrameric structure, leading to decreased expression levels and accelerated apoptosis of immature oocytes. All of the above demonstrate that TP63 with an active tetrameric structure is more prone to degradation, but more potent in function. This was consistent with our results (Fig. 2), where we also found that TP63 with a C-terminal truncation mutation showed a significant decrease in protein concentration but was a stronger inducer of apoptosis.

Our experiments showed that the *TP63* truncation mutation at the C-terminus could exacerbate cell apoptosis. In tumor-related studies, such as in Head and Neck Squamous Cell Carcinoma (HNSCC) cells, the TP63 C-terminal TID domain was found to inhibit apoptosis and promote tumor progression [56]. Further investigation revealed that the *TP63* C-terminal domain induced apoptosis mainly by activating pro-apoptotic genes on the mitochondrial membrane, including *BAX*, *BCL2L11*, *APAF1*, *RAD9*, and *DAP3*. It then binds specifically to the apoptotic receptors TRIP and DAP3, triggering a caspase cascade and inducing apoptosis. Knockdown of *TP63* results in decreased caspase expression and inhibits apoptosis [57]. In vivo, Lena et al. generated exon 13-truncated mice and found that the *TP63* C-terminal mutant activated the downstream target genes *PUMA* and *NOXA*, which directly or indirectly activated the BAX and BAK pathways, inducing cell stress and apoptosis [41, 58, 59]. However, in our study, we found no difference in the expression of *PUMA* and *NOXA* in cells with high expression of our *TP63* exon 13 truncation mutation. Interestingly, RNA sequencing revealed that the TP63 downstream target gene *CLCA2* was abnormally activated. RT-qPCR and western blotting also confirmed the significantly high expression levels of CLCA2. The Human Protein Atlas (https://www.proteinatlas.org/ENSG00000137975-CLCA2/tissue/ovary) indicates that the expression of CLCA2 (mRNA and protein) is not detected in the normal ovary, which was consistent with our results. This study suggests that an increase in *CLCA2* level may lead cells to enter the apoptotic process, and maintaining low level of *CLCA2* expression can prevent cell apoptosis (Fig. 6A and B).

Previous studies have shown that CLCA2 participates in cell proliferation, DNA damage, stress, apoptosis, adhesion, and invasion [60, 61], and thus participates in regulating inflammation and tumor progression. It was initially confirmed that CLCA2 is downregulated in breast cancer, and that its overexpression in breast cancer cells results in weakened tumorigenicity [62]. Subsequently, analysis of gene expression data from more than 1000 types of cancer tissues revealed that the expression of CLCA2 was significantly reduced in bladder cancer, esophageal cancer, lung cancer, high-grade nasopharyngeal carcinoma, colorectal cancer, lymphoma tissue, and prostate cancer [63–68]. The pH inside tumor cells is neutral or alkaline [69] which accelerates their immortality and invasiveness [70]. However, CLCA2 is an important component of the calcium-activated chloride channel (CACC) family [62], which regulates ion homeostasis, maintains a stable intracellular pH, changes the pH environment of tumor cells, and inhibits cancer progression [71]. At the cellular level, CLCA2 inhibits the WNT signaling pathway [72] or the ERK/JNK/p38-MAPK pathway to suppress epithelial-mesenchymal transition (EMT) [73, 74], thereby impeding tumor metastasis. The mechanism by which CLCA2 inhibits EMT was elucidated by Ramena et al., who proposed that it might involve the interaction of CLCA2 with EVA1/ZO-1 or β-catenin [72]. Meanwhile, the function of CLCA2 is also regulated by upstream factors. For example, abnormal activation of the p38/JNK-ATF2 pathway promotes high expression of CLCA3A2 and CLCA2 in the epidermal cells of mice with atopic dermatitis (AD) and the keratinocytes of patients with AD, inducing keratinocyte apoptosis [75].

CLCA2 is also involved in p53-mediated cell senescence, TP53 regulation of *CLCA2-*induced cancer cell cycle arrest, epithelial-mesenchymal transition (EMT)-mediated cancer progression [71], and cell apoptosis [60]. Interestingly, we found that high CLCA2 expression was associated with POI. An abnormally activated *CLCA2* promoter exacerbated cell apoptosis, while knocking down *CLCA2* alleviated this effect. Furthermore, the function of *CLCA2* was also regulated by TP63, and high expression of TP63 activated more *CLCA2*, resulting in a stronger induction of cell apoptosis. Therefore, we speculated that *CLCA2* may be a potential target for the treatment of POI. In our follow-up study, we will construct TP63-truncated mutant mice with POI and treat them with a si*CLCA2* nanodrug delivery system to observe whether it can improve ovarian function in mice.

The ATM-CHK1/CHK2-CK1-TP63 pathway is a classical pathway. In our study, we used an inhibitor of the upstream regulatory protein TP63, which significantly weakened its effect. The ATM kinase cascade reaction is triggered by DNA damage, particularly DSBs. Histone γ-H2AX can be phosphorylated at a lesion site and activate checkpoint kinases 1/2 (CHK1/2) [76–79]. CHK2 is phosphorylated at S582 [44] and collaborates with CHK1 to deliver signals to TP63; at the same time, the enhanced γ-H2AX signal in oocytes activates CK1, triggering the TP63 pathway, and induces oocyte apoptosis, autophagy, and cell cycle arrest [59], leading to oocyte exhaustion. This process can be effectively blocked using an ATM inhibitor that inhibits oocyte apoptosis [51]. Our previous studies have shown that CHK1 mutations cause meiotic arrest in fertilized eggs; however, treatment with a low-dose of CHK1 inhibitor results in ordered meiotic division and normal development of fertilized eggs [80]. Other studies have also shown that CHK2-TP63 signaling pathway is the main pathway in primordial follicle oocyte death [81]. BML-277, a CHK2 inhibitor, was found to act on mouse ovaries, and phosphorylation at *TP63* S582 was almost completely inhibited, with almost no tetramer formation, confirming the previously reported role of CHK2 in TP63 activation [42]. This study also compared the effects of ATM, CHK2, and CK1 inhibitors (data not shown), and found that the effect of the ATM inhibitor was the most significant and that a low dose of the ATM inhibitor could effectively weaken the regulation of TP63 on the *CLCA2* promoter and inhibit apoptosis. The combined effects of the ATM inhibitor and si*CLCA2* were even more significant. Therefore, the use of the ATM-CHK1/CHK2-CK1-TP63 pathway inhibitors weakened the regulation of TP63 truncation mutations on the *CLCA2* promoter and alleviated cell apoptosis. In the future, we will apply inhibitors of this pathway to TP63-truncating mutation mice to verify the precise and effective role of the ATM-CHK1/CHK2-CK1-TP63-CLCA2 signaling axis and discover novel targets for POI treatment.

## Conclusions

In summary, we identified a novel *TP63*-truncating mutation in one of 93 patients with sporadic POI. The TP63-truncated protein can induce cell apoptosis mediated by the direct transactivation of *CLCA2*. Silencing *CLCA2* using specific siRNAs or ATM inhibitors significantly decreased *CLCA2* expression and inhibited apoptosis induced by the overexpression of the TP63-truncated protein. Thus, our study proposes that silencing *CLCA2* may be a potential treatment for patients with POI harboring a *TP63*-truncating mutation. In the future, we will construct a *Tp63* pS551* truncated mutation POI mouse model to further explore the mechanism of *Tp63* truncated mutation causing POI. At the same time, we will treat the POI mouse with TP63 pathway inhibitors and *CLCA2* targeted drugs to explore whether POI symptoms can be relieved and provide more effective way for POI treatment.

### Supplementary Information

**Additional file 1:.** Supplemental Figure 1. The morphology of cells stained with Annexin-V and Hoechst33342 was observed under a fluorescence microscope with a scale bar of 25 μm. The siCLCA2 reduced the level of cell apoptosis induced by TP63-mut.

**Additional file 2:.** Supplemental Figure 2. The combination of ATMi and siCLCA2 inhibited the pro-apoptotic effect of the TP63-truncating mutation. This suggests that ATMi decreased the cell apoptosis induced by the TP63-mut protein by inhibiting CLCA2 expression. Scale bar = 25 μm.

**Additional file 3:.** Supplemental Figure 3.A. The DNA damage inducer Zeocin could increase the effect of TP63-truncated protein to induce more expression of CLCA2 with a dose-dependent manner.B. The DNA damage inducer Camptothecin (Topoisomerase inhibitor) could increase the effect of TP63-truncated protein to induce more expression of CLCA2 with a dose-dependent manner.

**Additional file 4.**

**Additional file 5.**

## Data Availability

The RNA-sequencing data has been uploaded to NCBI SRA database with accession number PRJNA970290 (https://www.ncbi.nlm.nih.gov/sra/PRJNA970290).

## Abbreviations

POI: Premature ovarian insufficiency
siRNA: small interfering RNA
ATM: Ataxia Telangiectasia Mutated
FSH: Follicle-stimulating hormone
TAD: Transcription activation domain
SAM: Sterile alpha motif domain
TID: Transcription inhibition domain
WES: Whole-exome sequencing
RNA-seq: RNA-sequencing
RT-qPCR: reverse transcription-quantitative polymerase chain reaction
WT: Wild-type
Mut: Mutant
NC: negative control
GO: Gene Ontology
ATMi: ATM inhibitor
EMT: Epithelial-mesenchymal transition
AD: Atopic dermatitis
