# Correction: TP63 truncating mutation causes increased cell apoptosis and premature ovarian insufficiency by enhanced transcriptional activation of CLCA2

**DOI:** 10.1186/s13048-024-01418-z

**Published:** 2024-04-29

**Authors:** Yali Fan, Shuya Chen, Chunfang Chu, Xiaodan Yin, Jing Jin, Lingyan Zhang, Huihui Yan, Zheng Cao, Ruixia Liu, Mingwei Xin, Lin Li, Chenghong Yin

**Affiliations:** 1 Central Laboratory, Beijing Obstetrics and Gynecology Hospital, Capital Medical University, Beijing Maternal and Child Health Care Hospital, Beijing, 100006 China; 2 Department of Gynecology, Beijing Obstetrics and Gynecology Hospital, Capital Medical University, Beijing Maternal and Child Health Care Hospital, Beijing, 100026 China; 3 Department of Traditional Chinese Medicine, Beijing Obstetrics and Gynecology Hospital, Capital Medical University, Beijing Maternal and Child Health Care Hospital, Beijing, 100026 China; 4 Department of Gynecological Endocrinology, Beijing Obstetrics and Gynecology Hospital, Capital Medical University, Beijing Maternal and Child Health Care Hospital, Beijing, 100026 China; 5 Department of Gynaecology and Obstetrics, Beijing Friendship Hospital, Capital Medical University, Beijing, 100050 China; 6 Department of Obstetrics, Beijing Obstetrics and Gynecology Hospital, Capital Medical University, Beijing Maternal and Child Health Care Hospital, Beijing, 100026 China; 7 Department of Laboratory Medicine, Beijing Obstetrics and Gynecology Hospital, Capital Medical University, Beijing Maternal and Child Health Care Hospital, Beijing, 100026 China

**Correction** **:** **J Ovarian Res 17, 67 (2024)**

**https://doi.org/10.1186/s13048-024-01396-2**

Following publication of the original article [1], the authors reported that there was an error in the additional file 1 wherein during compilation of the figure, images from incorrect group were inadvertently included in the panel of “TP63-WT+siCLCA2-1”. The correct images were shown below.

Incorrect additional file:


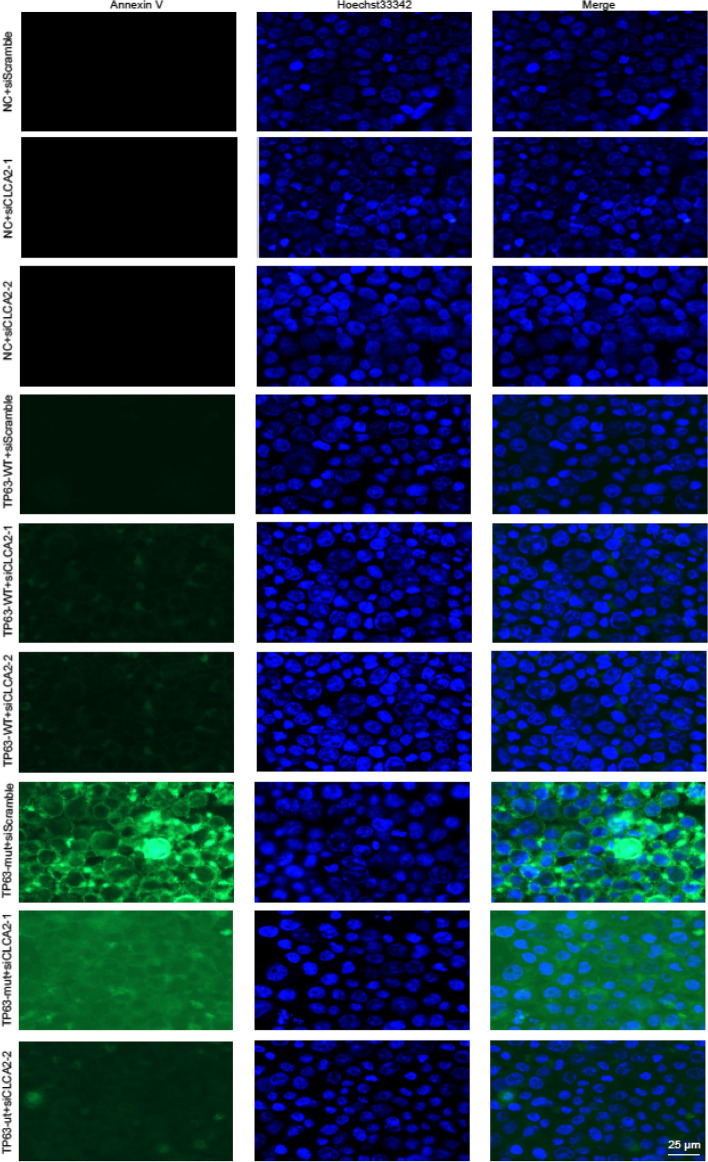


Correct additional file:


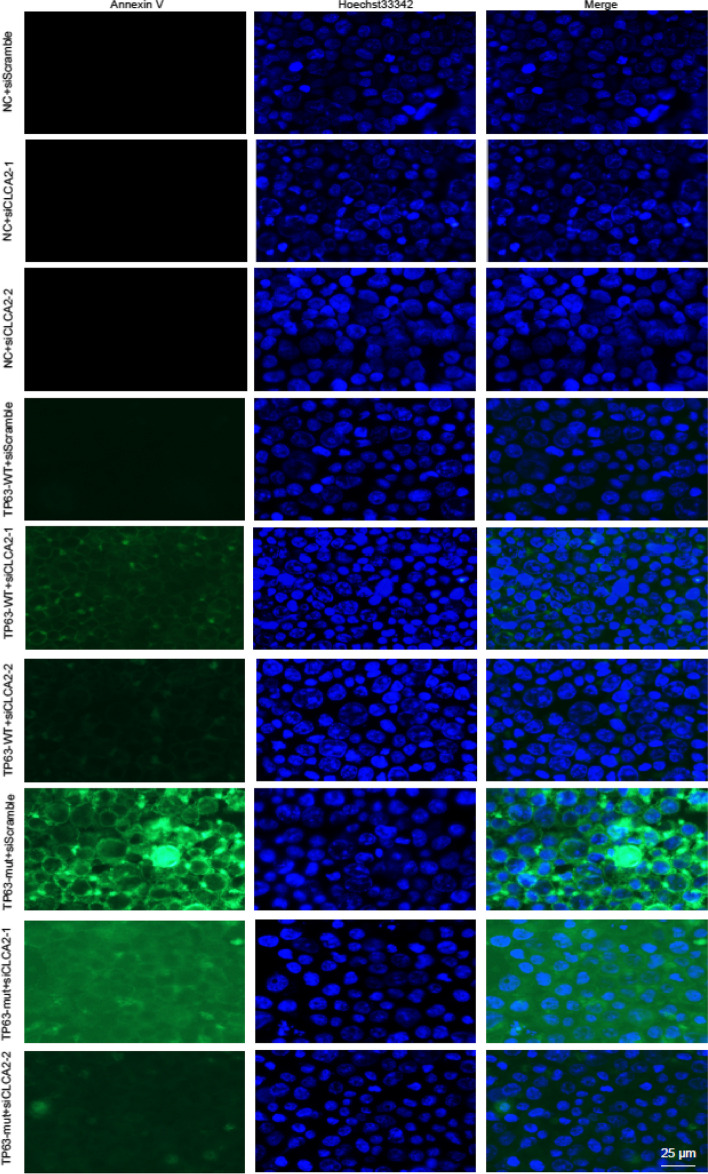


The original article has been corrected.

## References

[1] Fan Y, Chen S, Chu C (2024). TP63 truncating mutation causes increased cell apoptosis and premature ovarian insufficiency by enhanced transcriptional activation of CLCA2. J Ovarian Res.

