# Supplementary figures and images for: TP63 truncating mutation causes increased cell apoptosis and premature ovarian insufficiency by enhanced transcriptional activation of CLCA2
Source: J Ovarian Res. 2024 Mar 25;17:67. doi: 10.1186/s13048-024-01396-2 (PMC10962206; doi:10.1186/s13048-024-01396-2)

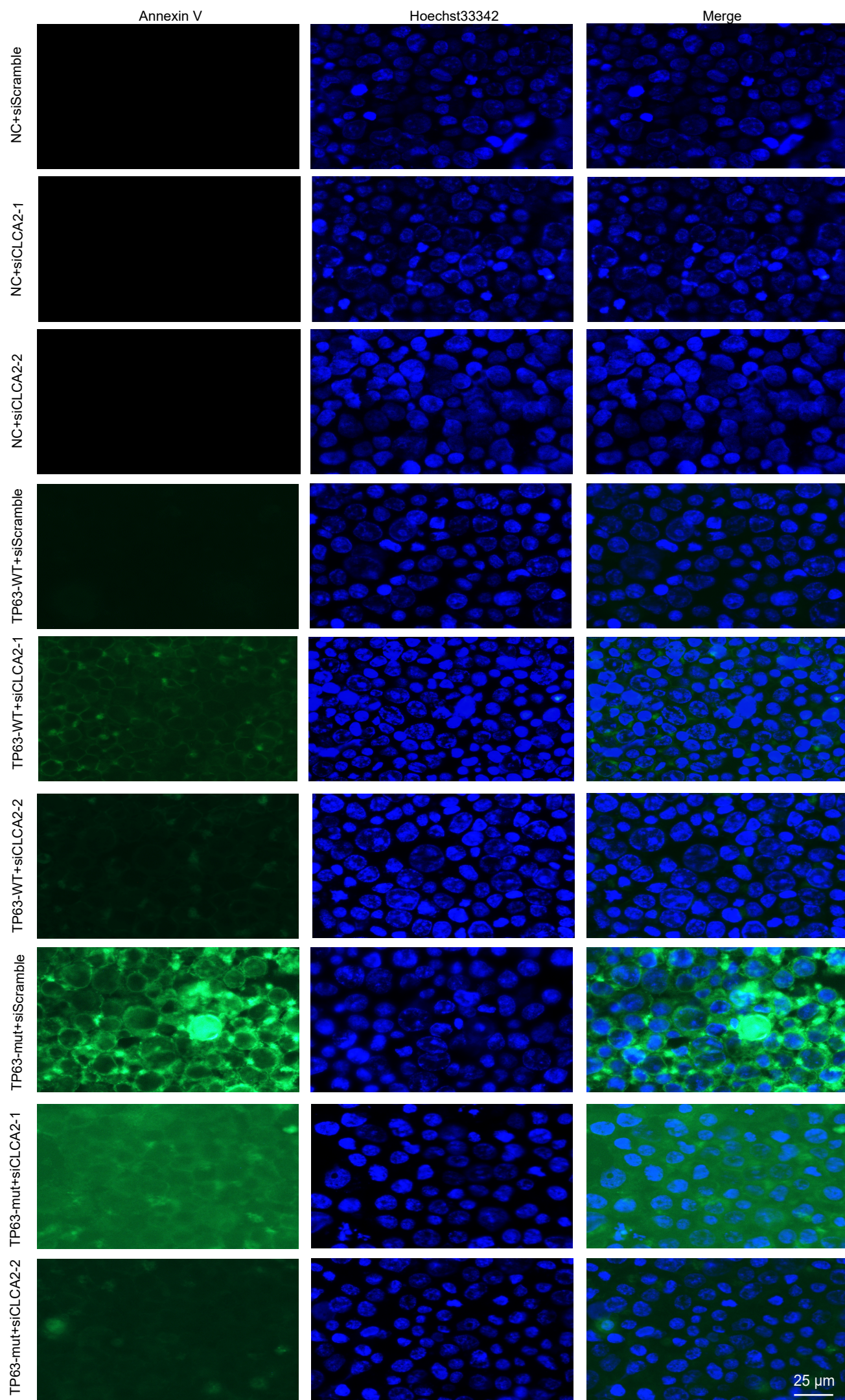

Supplement: Supplementary file 1 — Additional file 1:. Supplemental Figure 1. The morphology of cells stained with Annexin-V and Hoechst33342 was observed under a fluorescence microscope with a scale bar of 25 μm. The siCLCA2 reduced the level of cell apoptosis induced by TP63-mut. [file 13048_2024_1396_MOESM1_ESM.pdf]

A

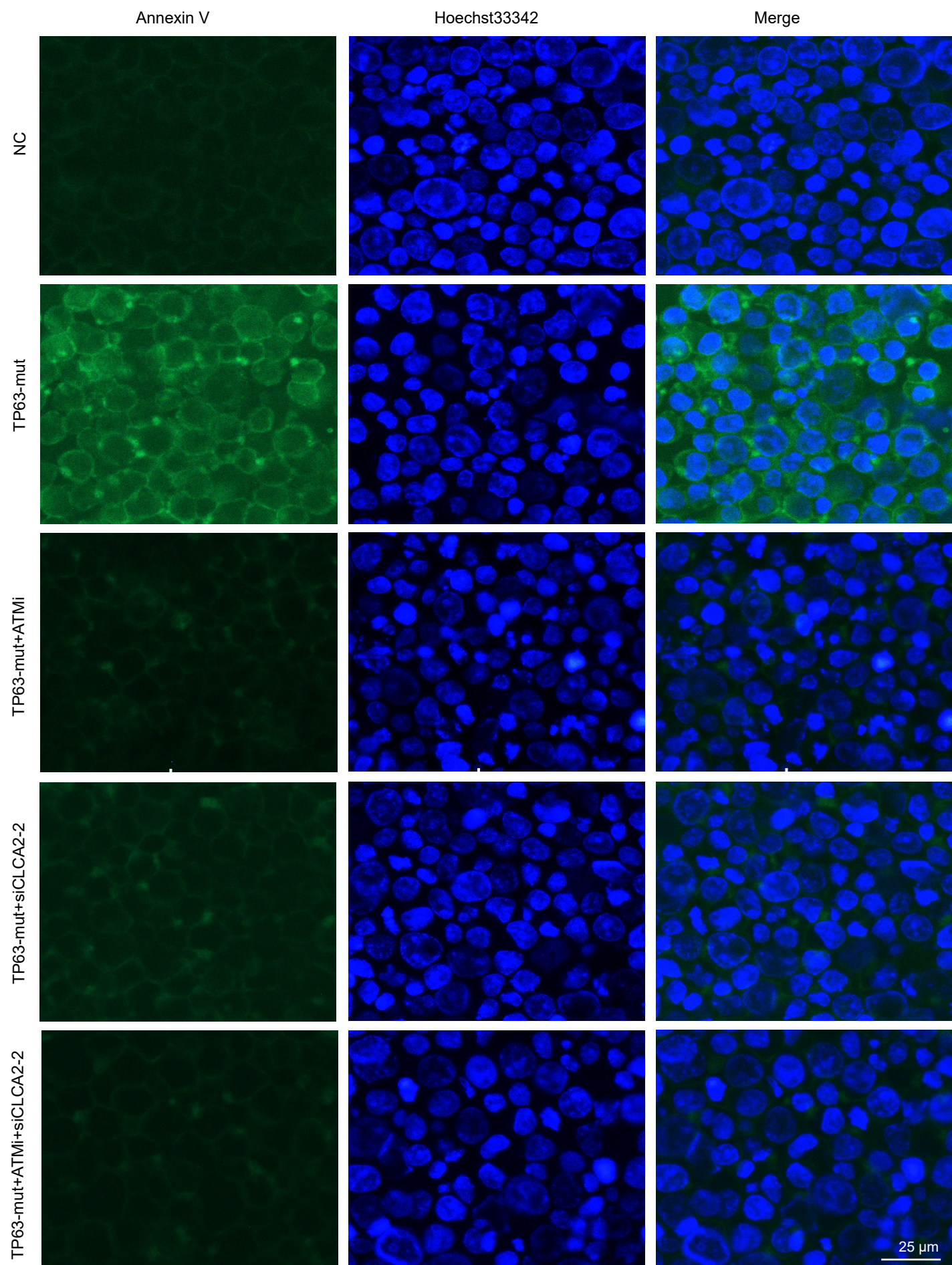

Supplement: Supplementary file 2 — Additional file 2:. Supplemental Figure 2. The combination of ATMi and siCLCA2 inhibited the pro-apoptotic effect of the TP63-truncating mutation. This suggests that ATMi decreased the cell apoptosis induced by the TP63-mut protein by inhibiting CLCA2 expression. Scale bar = 25 μm. [file 13048_2024_1396_MOESM2_ESM.pdf]

A

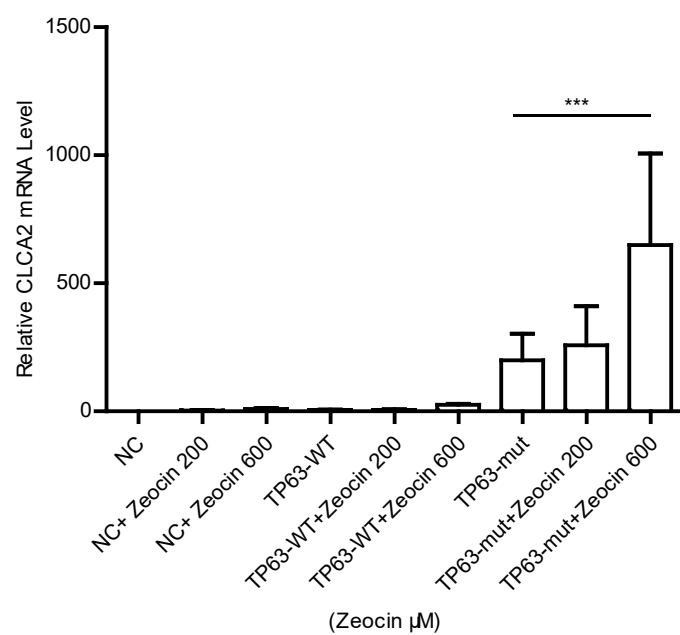

B

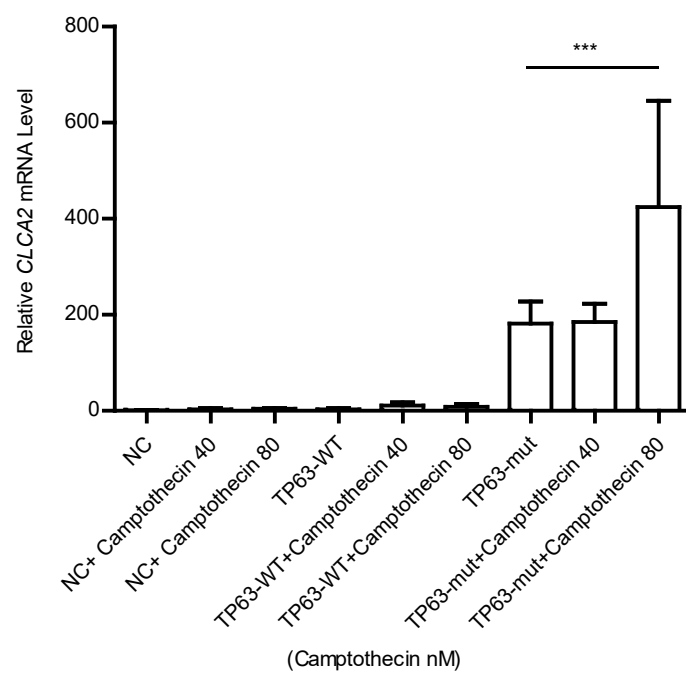

Supplement: Supplementary file 3 — Additional file 3:. Supplemental Figure 3.A. The DNA damage inducer Zeocin could increase the effect of TP63-truncated protein to induce more expression of CLCA2 with a dose-dependent manner.B. The DNA damage inducer Camptothecin (Topoisomerase inhibitor) could increase the effect of TP63-truncated protein to induce more expression of CLCA2 with a dose-dependent manner. [file 13048_2024_1396_MOESM3_ESM.pdf]
