# Supplementary material for: TP63 truncating mutation causes increased cell apoptosis and premature ovarian insufficiency by enhanced transcriptional activation of CLCA2
Source: J Ovarian Res. 2024 Mar 25;17:67. doi: 10.1186/s13048-024-01396-2 (PMC10962206; doi:10.1186/s13048-024-01396-2)
Supplement: Supplementary file 4 — Additional file 4. [file 13048_2024_1396_MOESM4_ESM.docx]

**Supplemental Table 1 The hormone levels of the POI patient**

| Date | FSH (IU/L) | LH (IU/L) | E2 (pmol/L) |
| --- | --- | --- | --- |
| Feb/2016 | 137.48 | 61.38 | 19.46 |
| Mar/2017 | 110.11 | 33.74 | 21.92 |

Note：FSH: follicle-stimulating hormone; LH: luteinizing hormone; E2：estradiol
