# Supplementary material for: TP63 truncating mutation causes increased cell apoptosis and premature ovarian insufficiency by enhanced transcriptional activation of CLCA2
Source: J Ovarian Res. 2024 Mar 25;17:67. doi: 10.1186/s13048-024-01396-2 (PMC10962206; doi:10.1186/s13048-024-01396-2)
Supplement: Supplementary file 5 — Additional file 5. [file 13048_2024_1396_MOESM5_ESM.docx]

**Supplemental Table 2. RT–PCR/RT-qPCR primers**

| **Gene** | **Forward primer (5′-3′)** | **Reverse primer (5′-3′)** | **Purpose** |
| --- | --- | --- | --- |
| *TP63-*gDNA | ACCATATGATAATCGCTGATCTGCA | TTCTATCAAAATGGATAATTGGGGG | Sanger Sequence |
| *TP63* | GGACCAGCAGATTCAGAACGG | AGGACACGTCGAAACTGTGC | RT-qPCR |
| *CLCA2* | GTGCATGGGATGTAATCACAGA | CAGCACTAAACAGACCACTTTGT | RT-qPCR |
| *ACTB* | GCACAGAGCCTCGCCTT | GTTGTCGACGACGAGCG | RT-qPCR |
| *TP63*-Exon12 | ATGGCTGGAGACATGAATGGA | - | Minigene assay |
| *TP63*-Exon13 | - | ATGATGAACAGCCCAACCTC | Minigene assay |
| *TP63*-Exon14 | - | ACACTGACTGTAGAGGCACTG | Minigene assay |
| *CLCA2*-promoter | atttGCTAGCtttaACTGATGGAGGAGGTTATGAA | ggccCTCGAGcgcgAGGTTTTAGAACACAAGATGAAGGA | Luciferase Assay |

**Supplemental Table 3. siRNA targeting sequences Gene**

| **siRNA** | **Sequences (5’-3’)** |
| --- | --- |
| siScramble | GUAAUUACAGCCUGUUG |
| siCLCA2-1 | UGACAAACCUUUCUACAUA |
| siCLCA2-2 | GGAAUUAUCACGUCUUACA |
| siCLCA2-3 | CAAUAGAUGAUCGAAAGUUG’ |
